# CHEMDNER system with mixed conditional random fields and multi-scale word clustering

**DOI:** 10.1186/1758-2946-7-S1-S4

**Published:** 2015-01-19

**Authors:** Yanan Lu, Donghong Ji, Xiaoyuan Yao, Xiaomei Wei, Xiaohui Liang

**Affiliations:** 1 School of Computer, Wuhan University, Wuhan 430072, China; 2 School of Public Health, Wuhan University, Wuhan 430072, China

**Keywords:** chemical named entity recognition, mixed conditional random fields, word clustering, deep learning

## Abstract

**Background:**

The chemical compound and drug name recognition plays an important role in chemical text mining, and it is the basis for automatic relation extraction and event identification in chemical information processing. So a high-performance named entity recognition system for chemical compound and drug names is necessary.

**Methods:**

We developed a CHEMDNER system based on mixed conditional random fields (CRF) with word clustering for chemical compound and drug name recognition. For the word clustering, we used Brown's hierarchical algorithm and Skip-gram model based on deep learning with massive PubMed articles including titles and abstracts.

**Results:**

This system achieved the highest F-score of 88.20% for the CDI task and the second highest F-score of 87.11% for the CEM task in BioCreative IV. The performance was further improved by multi-scale clustering based on deep learning, achieving the F-score of 88.71% for CDI and 88.06% for CEM.

**Conclusions:**

The mixed CRF model represents both the internal complexity and external contexts of the entities, and the model is integrated with word clustering to capture domain knowledge with PubMed articles including titles and abstracts. The domain knowledge helps to ensure the performance of the entity recognition, even without fine-grained linguistic features and manually designed rules.

## Background

Chemical and drug names are among the entity types most frequently searched in the PubMed database [1]. The recognition of such names are crucial for biomedical text processing tasks, e.g., detection of drug-protein interactions, adverse effects of chemical compounds and their associations to toxicological endpoints or extraction of pathway and metabolic reaction relations. Therefore, a high-performance named entity recognition system for chemical compound and drug names is necessary to ensure the performance of biomedical text processing tasks.

Chemical compound and drug name recognition was listed as a task in BioCreative IV [2], and it included two sub-tasks, i.e., indexing of documents with chemicals (chemical document indexing - CDI) and finding the mentions of chemicals in text (chemical entity mention recognition - CEM). Basically, this is a kind of named entity recognition (NER) tasks in natural language processing area. Similar tasks, such as gene and protein name recognition, have occurred in BioCreative II [3].

Several kinds of NER methods have been proposed in both the general domain [4-6] and the field of bioinformatics [7]. However, there are some new challenges in this task. First, the chemical compound and drug names may contain a number of symbols mixed with common words, e.g., '(22E,24R)-6β-methoxyergosta-7,22-diene-3β,5α-diol'. Another challenge is that the entity may consist of multiple phrases, e.g., 'C35-fluoro, C35-difluoro, and C35-trifluorosolamins', which is in fact a coordinate structure. Such examples pose a great deal of difficulties in recognition.

In this paper, we presented a method using mixed CRF models and word clustering. The method achieved the highest F-score of 88.20 % for CDI and the second highest F-score of 87.11% for CEM in BioCreative IV. Later, by using multi-scale word clustering based on a deep learning algorithm, we improved the F-score to 88.71% for CDI and 88.06% for CEM.

## Methods

**Figure 1:**
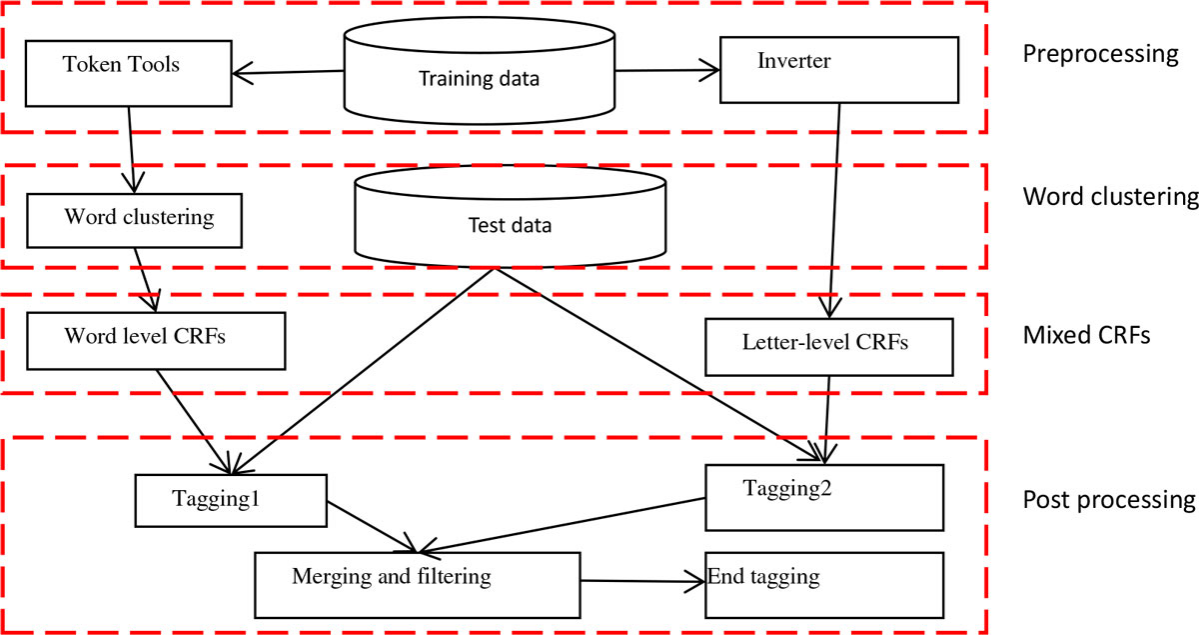
**gives the main framework of the system, which includes four parts: preprocessing, word clustering, mixed CRF and post processing**.

We considered the chemical compound and drug name recognition as a sequence labeling problem. CRF [8] is a classic and competitive method to solve this problem. CHEMDNER system mainly used CRF to recognize the chemical compound and drug names. Figure 1 gives the main framework of the system.

### Preprocessing

There are several kinds of symbols in chemical compound and drug names, as can be seen in examples, e.g., '[1,1'-biphenyl]-4,4'-diyldimethanamine', '(±)-methamphetamine' and 'catechin-[5,6-e]-4β-(3,4-dihydroxyphenyl)dihydro-2(3H)-pyranone'. In the PubMed corpus, some entities occurred with a very low frequency, so if recognizing the string as one unit in the sequence directly, the system would perform badly. One simple method might be to find the special characters, such as '[', 'β', 'Δ' and so on, and design the patterns for the strings, such as regular expressions "[a-z]*-\[\d,\d-[a-z]*\]-\d[β]-\(\d,\d-[a-z]*\)[a-z]*". However, it would be difficult to pre-define all such patterns manually.

To overcome such difficulties, we constructed a tokenizer with white space and the symbols listed in Table 1 to segment the sentences into tokens, so that we could obtain features, like prefix, suffix, digit, specific symbols and so on. We considered all non-alphabetic characters as the token symbol. During the training phase for character-level CRF, we took each character as a unit, and the character-level CRF model helped learn the internal features of the entity. In addition, we designed a character inverter to transform the corpus to a new one in the reversed order of the character, since previous studies showed that the F-score would be improved in the character level when training from right to left [12].

**Table 1:** Symbol tokens included in the tokenizer.

~ • ! @ # $ % ^ & * - = _ + ˉ ( ) [ ] { } ; ' : " , . / < > ×> < ≤ ≥ ↑ ↓ ← → • ′ ° ~ ≈ ? Δ ÷ ≠ | ‘ ’ “ ” §£ € \ 0 1 2 3 4 5 6 7 8

### Word clustering

Previous work showed that word clustering is a good feature for general named entity recognition [9]. Specifically, the clustering feature was used to improve gene and protein name recognition [12], and Turian et al. used the word representations based on Brown clusters to improve the named entity recognition in the field of news [11].

Our system used the Brown clusters that are created from a hierarchical word clustering algorithm [10]. 74,000 articles were downloaded from PubMed including titles and abstracts, using the entities in the training data as search keywords. The articles were then segmented with the tokenizer for further word clustering. We acquired 1,000 clusters from those articles and assigned each word a binary representation based on Huffman coding. Table 2 shows some examples. For example, the binary representation of the word "Confirms" is "10000110" which we used as the feature of the word.

**Table 2:** Binary representations for words.

Words	Binary representations
Confirms	10000110
Emphasis	10000110
Neighbourhoods	10101111010

**Figure 2:**
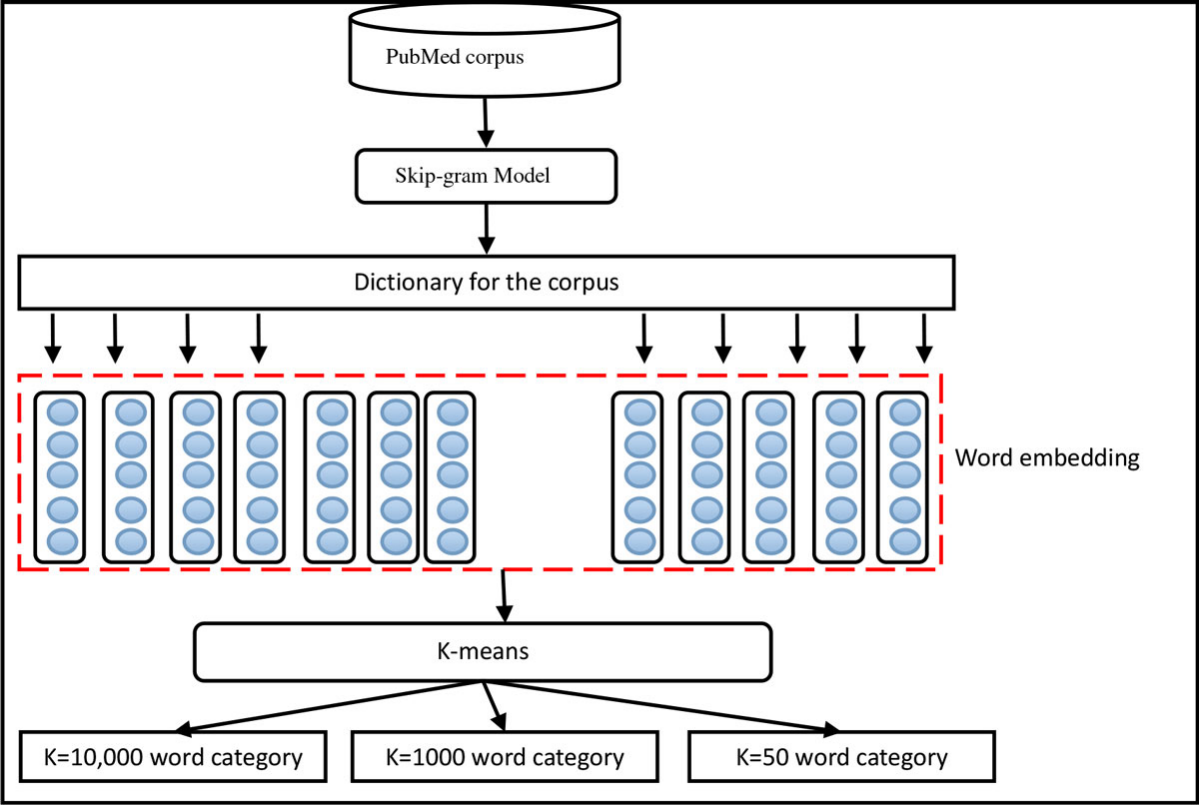
**First, we used a distributed word representations, i.e., Skip-gram model proposed by Mikolov et al.**[13]**, to obtain the word embedding**. Then we used K-means algorithm to acquire multi-level clusters based on the word embedding.

In addition, we tried multi-scale word clustering, as shown in Figure 2, to improve the performance of the system.

Finally, we used the multi-level clusters as word features in the CRF model. It was noted that the training time of the Skip-gram model is just a fraction of that required by Brown clustering. Thus, we could use more PubMed data to create the clusters. 400,000 PubMed articles were used to train the Skip-gram model with different size of clusters, and the model finally produced 50, 1000 and 10,000 clusters respectively.

### Mixed model

Now that the recognition of the chemical and drug names is seen as a sequence labelling problem, the objective is to identify the boundaries of the entities. In this sense, it becomes a problem of optimizing the model to fit the boundaries of the examples in the training data and improving the generalization ability of the model. In this study, we extended the CRF model to recognize chemical compound and drug names. In order to increase the model's scalability and flexibility, we utilized CRF++ in open source projects with extensible templates.

When training the corpus, we used the label set in the Table 3. Compared with the commonly used label set, {B, I, O}, this label set exhibited a more fine-grained distinction for the name components.

**Table 3:** Labels for the CRF model.

**No**.	Label	Explanation
1	B	beginning of a normal sequence

2	M	middle of a normal sequence

3	E	end of a normal sequence

4	S	single-token normal sequence

5	B_NER,	beginning of a NER sequence

6	M_NER	middle of a NER sequence

7	E_NER,	end of a NER sequence

8	S_NER	single-token NER sequence

In our study, we trained the CRF model in both the character and word level. Character-level features helped to model the internal structure of the chemical compound and drug names, and the word-level features to model the contexts of the names. Table 4 gives the character level features, where W_i _denotes the character in the i^th ^position in a sentence, and W_a_^b ^denotes the string of the characters from position a to b in a sentence.

**Table 4:** Character-based feature sets in CRFs model.

Character feature	W_i-4_, W_i-3_, W_i-2_, W_i-1_, W_i_, W_i+1_, W_i+2_, W_i+3_, W_i+4_
2-gram character feature	W_i-1_^i^, W_i_^i+1^

3-gram character feature	W_i-2_^i^, W_i-1_^i+1^, W_i_^i+2^

4-gram character feature	W_i-3_^i^, W_i-2_^i+1^, W_i-1_^i^,W_i_^i+1^

5-gram character feature	W_i-4_^i^, W_i-3_^i+1^, W_i-2_^i+2^, W_i-1_^i+3^, W_i_^i+4^

Table 5 gives the word level features in the model, where W_i _denotes the word in the i-th position in a sentence, W_a_^b ^denotes the string of words from position a to b in a sentence, C_i _denotes the category in the i-th position in a sentence, and C_a_^b ^denotes the category label string of the words from position a to b in a sentence.

**Table 5:** Word-based feature sets in CRFs model.

Word feature	W_i-2_, W_i-1_, W_i_, W_i+1_, W_i+2_
2-gram word feature	W_i-2_^i-1^, W_i-1_^i^, W_i_^i+1^, W_i+1_^i+2^

3-gram word feature	W_i-3_^i^, W_i-2_^i+1^, W_i-1_^i+2^, W_i_^i+3^

word cluster feature	C_i-2_, C_i-1_, C_i_, C_i+1_, C_i+2_

2-gram word cluster feature	C_i-2_^i-1^, C_i-1_^i^, C_i_^i+1^, C_i+1_^i+2^

3-gram word cluster feature	C_i-3_^i^, C_i-2_^i+1^, C_i-1_^i+2^, C_i_^i+3^

When training the character-based model, we trained the character-based model both from the left to the right and from the right to the left in each sentence. When training the word-based model, we combined the word features and cluster features. Moreover, we tried multi-scale cluster settings to explore their influence for the performance.

### Post processing

After the word-based and character-based models were trained, we merged the results in a heuristic method. Concretely, if the results from both models have only one same offset, we choose the one with higher confidence if the confidence difference is higher than 0.7; otherwise we keep the longer one. In addition, we filtered the entity if its confidence is lower than 0.0001. Finally, we refined the results using the strategies in Table 6. Take the row 1 as an example, it means that if the count of '(' is higher than that of ')' by 1 in the entity, and if to the right of the entity is ')', then we move the offset 1 token further to the right to include the symbol; otherwise, we delete the entity.

**Table 6:** Post-processing strategies.

Count(symbol) in entity	Symbol next to entity	Change offset
( = ) + 1	Right is )	Right + 1
( = ) - 1	Left is (	Left - 1
[ = ] + 1	Right is ]	Right + 1
[ = ] - 1	Left is [	Left - 1
{ = }+ 1	Right is }	Right + 1
{ = } - 1	Left is {	Left - 1

## Results and discussion

Both the data and evaluation tool were provided by BioCreative IV. The data includes 7,000 annotated PubMed data for training and 3,000 PubMed data for testing, and the evaluation tool reports precision, recall and F-scores. In addition to the data, we downloaded PubMed articles including titles and abstracts to produce word clusters. In the BioCreative IV evaluation, our system achieved the highest F-score of 88.20 % in the CDI task and the second highest F-score of 87.11% in the CEM task, which was only 0.28% lower than the highest F-sore of 87.39%.

### Baseline results

We chose the CRF models in both character and word levels without word clustering features as the baselines, Table 7 gives the baseline results. It shows that the character-based model performed better than word-based model in the two tasks, mainly because of the lower recall of the word-based model.

**Table 7:** Baseline result for CDI and CEM.

Baseline	CDI			CEM		
	
	Precision	Recall	F-score	Precision	Recall	F-score
**Character**	0.90706	0.85121	0.87825	0.91965	0.80450	0.85823

**Word**	0.90221	0.74536	0.81632	0.91774	0.70632	0.79827

### The results by our system in CHEMDNER task

Table 8 gives the results for our character-based and word based models. In the table, "Character+invert" means that we converted the sentence from right to left when training the CRF model in the character-based level, and the test data was handled in the same way. "Word+Brown" means that we segmented the corpus with the tokenizer, used Brown clustering algorithm to create 1,000 clusters, assigned each token a binary representation. Mixed system means that we merged the recognition results by the two models in character and word levels.

**Table 8:** CDI and CEM result by our system.

System	CDI			CEM		
	
	Precision	Recall	F-score	Precision	Recall	F-score
Character+invert	0.90766	0.85157	0.87872	0.91992	0.80482	0.85853

Word+Brown	0.89322	0.78393	0.83501	0.91069	0.74530	0.81973

Mixed system	0.87018	0.89408	0.88197	0.89105	0.85200	0.87109

Table 8 shows that the inverted corpus only achieved a little improvement. In comparison, word clustering features improved the recall rate more explicitly, which ensured that the F-scores were improved by 1.87% in CDI and 2.15% in CEM respectively. By the mixed model, the F-scores were further improved by 0.37% in CDI and 1.29% in CEM. The results indicate that the word clustering can improve the generalization ability of the CRF model. The reason may be that the word clusters, based on distributional similarity, can be seen as a kind of generalization over the individual words. Furthermore, such clusters provide more fine-grained distinction than commonly used parts-of-speech labels of the words.

### Improve system's result

After submission of the results, we made further improvement using multi-scale word clustering based on a skip-gram model. Table 9 gives the results.

**Table 9:** Result by skip-gram model and multi-scale word clustering.

System	CDI			CEM		
	
	Precision	Recall	F-score	Precision	Recall	F-score
A	0.87207	0.89328	0.88255	0.89243	0.85085	0.87115

B	0.86934	0.89861	0.88373	0.88985	0.85910	0.87420

C	0.86879	0.90256	0.88535	0.88840	0.86699	0.87756

D	0.86965	0.90351	0.88625	0.89017	0.86671	0.87828

E	0.87322	0.90007	0.88644	0.89263	0.86218	0.87714

F	0.86721	0.90782	**0.88705**	0.88725	0.87413	**0.88064**

G	0.86847	0.90387	0.88582	0.88845	0.86427	0.87619

H	0.86298	0.90818	0.88501	0.88373	0.87401	0.87884

**System configuration**: each configuration includes clustering method, the corpus size (S) and cluster number (N).

A: Brown clustering, S = 74 K, N = 1 K; B: Skip-gram model, S = 74 K, N = 1 K;C: Skip-gram model, S = 100 k, N = 1 K; D: Skip-gram model, S = 400 k, N = 1 K; E: Skip-gram model, S = 400 k, N = 10 k; F: Skip-gram model, Multi-level clustering, S = 400 k, N = 10 k,1 k,50; G: Skip-gram model, S = 2.4 M, N = 10 k; H: Skip-gram model, Multi-scale clustering, S = 2.4 M, N = 10 k,1 k,50.

Table 9 demonstrates the result was further improved by 0.12% for CDI and 0.3% for CEM by using Skip-gram to get the clustering features, as shown in System B. By increasing the corpus size or the cluster number, the performance appeared to have little change, as indicated in System C-E. Via multi-scale clustering of the words, system F achieved the F-score of 88.71% for CDI, which improved the F-score by 0.45%, the F-score of 88.06% for CEM, which improved the F-score by 0.95% compared to System A. The result indicates that word clustering features mainly improves the recall rate of the system.

On the other hand, we downloaded 2,400,000 articles randomly from PubMed, and tested the performance using the clusters based on the articles. System G-H in Table 9 shows that the result was not improved as expected. The reason may be that the articles were downloaded randomly from PubMed, not based on the entities annotated in the training data, and a lot of the articles were unrelated with them.

## Conclusions

In this work, we designed a mixed CRF model integrated with word clustering features to resolve the challenges in identifying chemical compound and drug names. The CRF model mixed a character-level with a word-level CRF model, and the clustering features were created from PubMed articles including titles and abstracts based on one-level or multi-level clusters. The system reached the F-score of 88.06% for the CEM task and 88.71% for the CDI task, which can be regarded as a very competitive result compared with the expected upper boundary, i.e., the agreement between two human annotators, i.e., 91% [3]. And our system can be downloaded from https://github.com/zuiwufenghua/biocreative_CHEMDNER.

The experiment showed that the mixing of the CRF models help to improve the performance, since the character-level CRF models the internal structures of the entities, while the word-level CRF helps to capture the external contexts of the entities. The experiment also showed that the clustering features, whether one-level or multi-level clusters, help to generalize the identified structures, thus improving the recall rates. This indicates that even though the automatically acquired word clusters only reflect course-grained domain knowledge, they can contribute to the named entity identification.

With the present task setting as a sequence labeling problem, there would be several future work worth further investigation. First, a lot of new machine learning techniques have been proposed to solve the sequence labelling problem successfully. For example, Mann et al. [18] presented a semi-supervised CRF to improve sequence segmentation and labelling. Yu et al. [19] proposed a deep-structured CRF for sequence labelling. Collobert et al. [20] came up with a deep learning architecture for NLP, which can be used to label the sequence. In future, we will apply such methods to chemical and drug name identification.

On the other hand, features would be crucial for the performance of such sequence labeling tasks, so another future work would be exploring various structural, contextual or categorical features to enrich the CRF models. In addition, future work also includes extending such methods to other labeling tasks in biomedical domain, e.g., gene names or protein-protein relations, etc.

## Competing interests

The authors declare that they have no competing interests.

## Authors' contributions

YL, DJ and XL conceived the study, YL carried out the implementation. YL, DJ, XY and XW wrote the manuscript. All contributed to the intellectual evolution of this project. All authors have read and approved the final manuscript.

## Declarations

Publication of the work is funded by the following projects: NSF projects of China No.61133012, No. 61373108, No.61202304, Social Science Foundation project No. 11&ZD189, and Hubei NSF project 2012FFA088.

This article has been published as part of *Journal of Cheminformatics *Volume 7 Supplement 1, 2015: Text mining for chemistry and the CHEMDNER track. The full contents of the supplement are available online at http://www.jcheminf.com/supplements/7/S1.
